# Mental workload according to sociodemographic and occupational characteristics of professionals in charge of health programs

**DOI:** 10.47626/1679-4435-2024-1245

**Published:** 2025-01-31

**Authors:** Carolina Elena Luengo-Martínez, Jaime Sebastián Castro-Zapata, Nicole Constanza Guíñez-Pérez

**Affiliations:** 1 Vice Rectorate for Research and Graduate Studies, Universidad del Bío-Bío, Chillán, Ñuble, Chile

**Keywords:** working conditions, health personnel, primary health care, condiciones de trabajo, personal de salud, atención primaria de salud

## Abstract

**Introduction:**

Primary health care services are focused on promotion, prevention, and rehabilitation, in addition to implementing various health programs. Mental workload is defined as mental, cognitive, or intellectual demands experienced by workers during their workday.

**Objectives:**

To describe mental workload according to sociodemographic and occupational characteristics of professionals responsible for health programs in primary care facilities in the Ñuble region, Chile.

**Methods:**

A descriptive, cross-sectional quantitative study was conducted with a sample of 69 professionals. Data were collected through surveys assessing sociodemographic and occupational characteristics, workplace environmental conditions, and the *Escala Subjetiva de Carga Mental de Trabajo* (Subjective Mental Workload Scale), validated for use in Chile.

**Results:**

The mental workload was moderate-to-high, with a mean score of 3.68. The dimensions “cognitive demands and task complexity” and “task characteristics” presented the highest mean scores, while “time organization” and “work pace” showed the lowest mean scores among the variables studied.

**Conclusions:**

Younger workers with fixed-term contracts, who perceived their work environment conditions as highly inadequate and were in charge of more than 16 people presented the highest levels of mental workload.

## Abstract (es)

**Introducción:**

La atención primaria en salud entrega prestaciones enfocadas en promoción, prevención y rehabilitación, además de desarrollar diversos programas de salud. El concepto de carga mental de trabajo se define como los requisitos mentales, cognitivos o intelectuales que enfrentan los(las) trabajadores(as) durante su jornada laboral.

**Objetivos:**

Describir la carga mental de trabajo según características sociodemográficas y laborales en profesionales encargados de programas de salud de establecimientos de atención primaria de la región de Ñuble.

**Métodos:**

Se utilizó un diseño cuantitativo descriptivo de corte transversal con una muestra de 69 profesionales, y se recopiló información a través de cuestionarios sobre características sociodemográficas y laborales, condiciones ambientales de trabajo y la Escala Subjetiva de Carga Mental de Trabajo validada en Chile.

**Resultados:**

La carga mental de trabajo fue media-alta, con un promedio de 3,68 puntos. Las dimensiones de “demandas cognitivas y complejidad de la tarea” y “características de la tarea” presentaron los promedios más altos, mientras que las dimensiones de “organización temporal” y “ritmo de trabajo” obtuvieron las medias más bajas según las variables estudiadas.

**Conclusiones:**

Se evidenció que los(las) trabajadores(as) jóvenes, con contrato a plazo fijo, que percibían condiciones ambientales de trabajo muy inadecuadas y tenían a su cargo más de 16 personas fueron los que presentaron mayor carga mental de trabajo.

## INTRODUCTION

Globally, primary health care (PHC) serves as the entry point into the health care system, focusing on promotion, prevention, and rehabilitation.^1,2^ Its origins date back to the 1978 Alma-Ata Conference, where it was first defined.^2^ In Chile, PHC encompasses specific functions^3^ but is generally aimed at supporting individuals across their entire life cycle, from birth and growth to rehabilitation and reproductive health. It also promotes self-management through a family and community health approach.^4^ PHC in Chile develops various health programs that focus on the community and different life stages, from childhood to aging, in accordance with national ministerial regulations.

To achieve the objectives of PHC, professionals working at this level must take on managerial roles in addition to their clinical work, which involve continuous contact with individuals and the community. Within this high-demand work environment, PHC workers often experience mental workload (MWL) with a tendency toward overload due to the multiple occupational factors faced during their shifts.^5^

Mental load is a concept that has no specific definition. However, Hacker^6^ has approached it from two perspectives: first, by considering the task characteristics and the tools available to address it; and second, by examining the inherent demands of the task itself. Díaz et al.^7^ conceptualized MWL as a multidimensional construct resulting from the interaction between three aspects: task demands, individual characteristics, and situational characteristics.

This mental load can impact the worker’s health,^8^ impair work performance, reduce productivity,^9^ and compromise the quality of health care services provided.

In this regard, studies have shown that an increase in mental load can negatively impact workers’ performance,^10^ ultimately affecting their health status.^11^ This is particularly evident in the psychological domain, where exceeding limits can lead to behaviors that develop over time, such as a hostile work environment, mistreatment among staff, and even mental health disorders, which may result in extended medical leave. These issues disrupt the functioning of health care centers, leading to harm to patients as the working hours of various professionals are reduced due to absenteeism. Research has shown that workers with high mental load are more likely to experience work-related absences.^12^ It is important to note that individuals who do not experience high levels of mental load tend to perform their work more effectively,^13^ which highlights mental load as a psychosocial risk factor in the workplace.

For these reasons and given the lack of evidence from studies analyzing MWL in this group of professionals, the present study aimed to describe MWL based on sociodemographic and occupational characteristics of health program professionals working in PHC facilities in the Ñuble region of Chile.

## METHODS

This quantitative, descriptive cross-sectional study included a sample of 69 professionals from the *Programas de Salud del Adulto* (Adult Health Programs), *Programa de Salud Cardiovascular* (Cardiovascular Health Program), *Programa de Personas Mayores* (Older Adults Program), and *Programa de Atención Domiciliaria a Personas con Dependencia Severa* (Home Care Program for Individuals with Severe Dependency). Inclusion criteria were: a) health professionals, either primary or substitute, in charge of a health program at a primary care facility in the Ñuble region for at least 3 months; and b) professionals performing both clinical and administrative tasks. Exclusion criteria were: a) health professionals working remotely; and b) health professionals on medical leave, permitted leave, or in COVID-19 quarantine at the time of the instrument application. Permission to conduct the research with staff was obtained from the relevant management personnel, and the study was approved by the Research Ethics Committee of the Hospital Clínico Herminda Martín.

Data collection was conducted in person at PHC facilities in the Ñuble region from February to March 2023. The importance of the study was emphasized to management teams, and participants were sensitized through an explanatory session detailing the topic, objectives, and relevance of the study. Workers were invited to participate voluntarily, with informed consent obtained. The following instruments were used: a survey on sociodemographic and occupational characteristics developed by the authors and a questionnaire on workplace conditions. The latter, part of the *Escala Subjetiva de Carga Mental de Trabajo* (ESCAM, Subjective Workload Assessment Scale), evaluates factors such as temperature, lighting, noise, space, and hygiene. Responses were rated on a Likert scale from 1 (very inadequate) to 5 (very adequate).^14^

The ESCAM, developed by Rolo et al.^15^ in 2009, consists of 20 items categorized into five dimensions. Each item is rated on a Likert scale from 1 (low mental load) to 5 (high mental load). The MWL is assessed across the following dimensions: “cognitive demands and task complexity”, “task characteristics”, “time organization”, “work pace”, and “health consequences”. In 2014, Ceballos-Vásquez et al.^16^ validated the instrument for use in Chile, demonstrating high reliability through internal consistency (Cronbach’s alpha = 0.73) and adequate validity, making it suitable for the Chilean population. For this study, the instrument showed a reliability score of 0.74 (Cronbach’s alpha), which is considered close to acceptable.

In descriptive analysis of the sociodemographic and occupational factors, summary measures (central tendency and dispersion) were used for quantitative variables, and absolute and relative frequencies were used for qualitative variables. Data analysis was performed with SPSS version 29.0.

## RESULTS

The mean score for MWL in the sample was 3.68, which indicates a moderate-to-high MWL, considering that 1 represents low mental load and 5 represents high mental load. The dimensions “cognitive demands and task complexity” and “task characteristics” stood out, with mean scores close to 4 points, indicating a high MWL. In contrast, the dimension “work pace” had the lowest mean score, 3.23, which still indicates a moderate-to-high MWL (Table 1).

**Table 1:** Mental workload of the sample

	Result 1 Minimum	Result 2 Maximum	Result 3 Mean ± SD
Mental workload	2.87	4.70	3.68±0.44
Cognitive demands and task complexity	2.67	5.00	4.01±0.49
Task characteristics	2.25	5.00	4.04±0.56
Time organization	1.00	5.00	3.41±0.80
Work pace	1.00	5.00	3.23±0.92
Health consequences	1.50	5.00	3.74±0.80

Regarding the sociodemographic variables of the sample, nearly 50% (n = 31) were individuals aged 26 to 31 years. Overall, 84.1% (n = 58) were women, and 73.9% (n = 51) were in a relationship.

There were no data available on the type of employment contract. In the sample, 26.7% (n = 31) oversaw the Cardiovascular Health Program, and 29% (n = 20) were in charge of three programs.

Regarding the working environmental conditions, 33.3% (n = 23) of the sample perceived the temperature as intermediate, 49.3% (n = 34) perceived the lighting as adequate, and 39.1% (n = 27) perceived the noise levels as intermediate. As for the workspace, 24.6% (n = 17) rated it as intermediate, adequate, or very adequate. Lastly, 43.5% (n = 30) considered the hygienic conditions to be adequate.

Among the professionals, 56.5% (n = 39) supervise between zero and seven people, while 46.4% (n = 32) oversee fewer than 1,000 individuals in the program(s) under their responsibility.

Those in charge of programs and aged 26 to 31 years reported a mean MWL of 3.75, indicating moderate-to-high MWL, slightly higher than that of other age groups. Both sexes presented moderate-to-high MWL. Similarly, both individuals with a partner and those without a partner showed moderate-to-high MWL (Table 2).

**Table 2:** Mental workload according to sociodemographic variables

Variable	n	Result 1 Mean ± SD of mental workload
Age (years)		
26-31	31	3.75±0.41
32-37	19	3.73±0.49
38-43	10	3.42±0.39
44-49	3	3.59±0.65
50-55	5	3.70±0.37
≥ 56	1	3.68±0.00
Sex		
Male	11	3.65±0.41
Female	58	3.69±0.45
Relationship status		
With a partner	51	3.66±0.45
Without a partner	18	3.75±0.41

Regarding weekly working hours, workers with a 33-hour workweek exhibited a high mental load. Conversely, in terms of type of contract, all three groups demonstrated moderate-to-high MWL. A similar situation was observed in terms of the number of programs in charge, the number of personnel supervised (Table 3), and working environmental conditions (Table 4). No significant differences in MWL were observed based on the population under supervision (Table 3).

**Table 3:** Mental workload according to work-related variables

Variable	n	Result 1 Mean ± SD of mental workload
Weekly working hours		
33	1	4.03±0.00
44	67	3.69±0.43
50	1	2.98±0.00
Type of contract		
Permanent contract	37	3.62±0.43
Fixed-term contract	28	3.78±0.44
Freelance contract	4	3.65±0.46
Number of programs overseen		
1	13	3.54±0.38
2	13	3.77±0.40
3	20	3.67±0.48
4	11	3.71±0.53
5	10	3.77±0.43
6	2	3.72±0.28
Number of staff supervised		
0-7	39	3.68±0.46
8-15	18	3.65±0.44
≥ 16	12	3.75±0.35
Population under program supervision (people)		
< 1,000	32	3.69±0.44
1,000-3,000	24	3.68±0.41
> 3,000	18	3.67±0.50

**Table 4:** Mental workload according to working environmental conditions in the sample

	Working environmental conditions				
	Very inadequate x¯ ± SD	Inadequate x¯ ± SD	Intermediate x¯ ± SD	Adequate x¯ ± SD	Very adequate x¯ ± SD
Temperature at workstation	n = 5	n = 12	n = 23	n = 19	n = 10
Mental workload	3.95±0.35	3.75±0.50	3.80±0.32	3.60±0.50	3.37±0.34
Lighting at workstation	n = 1	n = 5	n = 11	n = 34	n = 18
Mental workload	3.63±0.00	3.75±0.45	3.84±0.30	3.69±0.49	3.57±0.40
Noise at workstation	n = 5	n = 11	n = 27	n = 15	n = 11
Mental workload	3.75±0.39	3.87±0.43	3.70±0.40	3.69±0.48	3.41±0.45
Workspace at workstation	n = 7	n = 11	n = 17	n = 17	n = 17
Mental workload	3.98±0.29	3.80±0.55	3.61±0.39	3.77±0.43	3.49±0.39
Hygienic conditions at workstation	n = 3	n = 7	n = 16	n = 30	n = 13
Mental workload	3.96±0.34	3.73±0.50	3.74±0.37	3.73±0.45	3.42±0.40

Table 5 shows that the “task characteristics” dimension presents a high MWL in those responsible for the Adult Health Program, with a mean value of 4.13 points, the Cardiovascular Health Program, with a mean value of 4.08 points, and the Older Adults Program, with a mean value of 4.05 points. On the other hand, professionals in charge of the Home Care Program for People with Severe Dependency show a high MWL in the “cognitive demands and task complexity” dimension, with a mean value of 4.09 points. Overall, all program managers demonstrate a moderate-to-high MWL.

**Table 5:** Mental workload according to program(s) overseen by the sample

Mental workload dimensions	Program(s) overseen			
	AHP n = 27	CHP n = 31	OAP n = 29	HCPSD n = 29
	x¯ ± SD	x¯ ± SD	x¯ ± SD	x¯ ± SD
Mental workload	3.69±0.45	3.68±0.45	3.70±0.42	3.69±0.47
Cognitive demands and task complexity	3.96±0.54	3.94±0.51	4.02±0.47	4.09±0.50
Task characteristics	4.13±0.61	4.08±0.65	4.05±0.64	4.03±0.48
Time organization	3.40±0.94	3.39±0.97	3.47±0.74	3.45±0.74
Work pace	3.23±0.90	3.27±0.85	3.26±0.81	3.15±1.06
Health consequences	3.71±0.84	3.73±0.88	3.68±0.81	3.75±0.78

AHP = Adult Health Program; HCPSD = Home Care Program for Individuals with Severe Dependency; OAP = Older Adults Program; CHP = Cardiovascular Health Program.

## DISCUSSION

The mean mental load of the 69 respondents was 3.68, indicating a moderate-to-high MWL. This aligns with the findings of Espinoza,^17^ who reported in 2020 a mean MWL of 3.69 among health care workers in both the private and public sectors. According to Delliaux et al.,^8^ this could impact workers’ cardiovascular health. Additionally, the results highlight that high MWL can negatively affect employee performance, reducing labor productivity,^9^ decreasing the effectiveness of care, and increasing absences, which in turn reduces the hours available for patient care in health care centers.

Regarding work-related variables, it is noteworthy that 97.1% of the professionals worked 44 hours per week, indicating that the 8:00 AM to 5:00 PM schedule is the most common in PHC. Notably, the literature review did not identify any studies that specifically addressed this variable among primary care professionals.

In relation to working environmental conditions, professionals perceived them as ranging from intermediate to very adequate for temperature, lighting, noise, space, and hygiene. This is particularly relevant, as these are health care centers that must meet minimum standards to provide quality and safe services to both external and internal users. In addition, there is a direct relationship between working environmental conditions and job performance, suggesting that better environmental conditions lead to improved worker performance.^18-20^

In terms of MWL based on sociodemographic characteristics, the highest frequency was found among individuals aged 26 to 31, with this group presenting a moderate-to-high MWL.

In relation to sex, women had a mean of 3.69 points, slightly higher than that of men, indicating that both sexes experienced a moderate-to-high MWL. This aligns with the findings of Alcántara et al.^5^ and Fernández et al.,^21^ who studied mental load in the Spanish population. They found that, although women had a higher mental load, this difference could not necessarily be attributed to the sex variable.

The difference in the mean MWL between professionals who were in a relationship and those who were not was 0.09 points, similar to the 0.01 points reported in a 2017 study.^5^ It is worth noting that the trend toward a moderate-to-high MWL is maintained in the sample.

With respect to MWL based on work-related variables, a significant proportion of individuals in the sample worked 44 hours per week. It is noteworthy that the individual who reported working 33 hours per week had a higher mean mental load than those in the other two categories, while the individual working 50 hours had a mean of 0.71 points lower compared to those working 44 hours. Given the small number of individuals in the 50-hour group, it cannot be determined whether this is a trend or an isolated case.

Regarding type of contract, a moderate-to-high MWL was observed across all three types of contracts, which could indicate that, although the type of contract may provide a sense of greater economic stability, it does not prevent a high MWL.

In relation to the programs they were in charge of and the number of programs overseen, the MWL of those in charge of the addressed programs is similar. It is also noteworthy that individuals in charge of the Adult Health Program, the Cardiovascular Health Program, and the Older Adults Program share the “task characteristics” dimension, which presents the highest MWL. This indicates that these workers are primarily exposed to interruptions and the execution of multiple tasks simultaneously, requiring a higher level of concentration. This finding is significant, given that these programs are conducted entirely at the health care center. Conversely, the Home Care Program for Individuals with Severe Dependency presents a high MWL in the “cognitive demands” dimension, which could be due to the fact that nearly all clinical activities are performed at the patients’ homes, requiring a higher level of memorization and mental effort.

Conversely, no significant differences in MWL were observed based on the number of programs overseen, as all presented a moderate-to-high MWL. However, as mentioned earlier, managing a large number of programs can result in a moderate-to-high MWL, as it entails completing a greater number of tasks within a limited time.

Regarding working environmental conditions, MWL was not directly affected by factors such as temperature, lighting, noise, space, and hygienic conditions. This contrasts with the findings of Ceballos, who states that working environmental conditions are negatively related to factors such as “time organization”, “work pace”, and “task characteristics”. In other words, when workers perceive inadequate working environmental conditions, their perception of MWL increases.^22^ This can be explained by the fact that Ceballos conducted his study in an intrahospital environment, where there are slight differences in noise and space conditions at the workplace compared to the results of the present study.

Regarding the number of staff under supervision, no significant differences were observed in the overall MWL, as all exhibited a moderate-to-high MWL. Finally, in relation to MWL based on the population under the responsibility of the professional(s), it did not vary significantly across different ranges, with all showing a moderate-to-high MWL.

One of the limitations of the present study is the limited research addressing the issue of mental load among PHC professionals in Chile. Based on this, it is suggested that further research be conducted at this level of care to achieve greater representation of certain variables, such as number of working hours. Similarly, further studies on this topic are encouraged, aiming to advance the analysis of the relationship between the presented variables and MWL.

## CONCLUSIONS

The professionals in charge of health programs at primary care facilities in the Ñuble region are primarily young adults, women, and individuals with partners, all of whom exhibit a moderate-to-high MWL.

In line with the general objective, it is concluded that young workers with fixed-term contracts, who perceive very inadequate working environmental conditions, and who supervise more than 16 people exhibit higher MWL. No significant differences in MWL are observed based on sex, relationship status, the program they oversee, the number of programs overseen, or the population under their care.
